# Dealing with flawed items in examinations: Using the compensation of disadvantage as used in German state examinations in items with partial credit scoring

**DOI:** 10.3205/zma001195

**Published:** 2018-11-15

**Authors:** Andreas Möltner

**Affiliations:** 1 Medizinische Fakultät Heidelberg, Kompetenzzentrum für Prüfungen in der Medizin, Heidelberg, Germany

**Keywords:** examination, IMPP

## Abstract

In the written part of German state exams, multiple choice questions which inherit a flaw are not always excluded but taken into consideration when awarding a grade to any individual candidate, if this is advantageous to the candidate. This process of elimination and compensation of disadvantage in flawed questions, as applied by the Institut für medizinische und pharmazeutische Prüfungsfragen (IMPP), can lead to *different* *items* being taken into consideration in the grading *in individual cases* and to the application of *different pass and grade boundaries in individual cases*.

This procedure is applied here to examinations containing items with partial credit scoring. A simple numerical rule can be applied which can be used to decide whether a flawed item has be taken into account in the grading procedure in order to achieve a pass or a particular grade for a candidate. This rule – as in the state examinations – must take account of the individual number of scores achieved in the flawed item and, if the relative pass threshold ("automatic adjustment clause") is applied, the average score achieved in the item by all candidates. In addition, however, it also includes the grade boundaries.

## Zusammenfassung

Sind in den schriftlichen Teilen der deutschen Staatsexamina Aufgaben enthalten, die inhaltlich oder formal fehlerhaft sind, werden diese nicht generell ausgeschlossen, sondern bei der Benotung eines einzelnen Prüfungsteilnehmers dennoch berücksichtigt, wenn dies für diesen Teilnehmer *vorteilhaft* ist. Dieses Verfahren von Elimination und Nachteilsausgleich bei fehlerhaften Fragen, wie es vom Institut für medizinische und pharmazeutische Prüfungsfragen (IMPP) angewandt wird, kann dazu führen, dass bei einer Prüfung* individuell unterschiedliche Aufgaben* in der Bewertung berücksichtigt werden sowie* individuell unterschiedliche Bestehens- und Notengrenzen* gelten.

Dieses Vorgehen wird auf Prüfungen übertragen, die Aufgaben enthalten, bei denen Teilpunkte vergeben werden. Es lässt sich ein einfaches numerisches Kriterium angeben, mit dem entschieden werden kann, ob eine fehlerhafte Aufgabe zum Erreichen der Bestehens- oder einer Notengrenze bei einer Prüfungsteilnehmerin oder einem Prüfungsteilnehmer für die Bewertung zu berücksichtigen ist oder nicht. In dieses Kriterium geht – wie bei den Staatsexamina – die individuell erreichte Punktzahl bei der fehlerhaften Aufgabe und bei Anwendung der relativen Bestehensgrenze („Gleitklausel“) die mittlere erreichte Punktzahl aller Teilnehmer bei der Aufgabe ein. Darüber hinaus beinhaltet es aber auch noch die Notengrenzen.

## 1. Background

### 1.1. Flawed items in examinations

Despite the careful review of examination items *before the examination*, it is almost impossible to avoid the discovery *after the examination* that individual items contain errors of content or form, for example that in a Type A multiple-choice item ("one out of five") in fact two possible answers must be acknowledged as being correct. An item such as this is considered to be "flawed" because, contrary to convention, it permits more than one single correct answer.

Those responsible for examinations within the faculties are therefore faced with the problem of needing to develop a legally sound procedure for dealing with flawed items. Study regulations and examination guidelines frequently contain insufficient directives regarding this aspect, which is why the approach of the Institut für medizinische und pharmazeutische Prüfungsfragen (IMPP), which is seen as legally sound, has been or is soon to be adopted by faculties for their own examinations in some places.

This is possible as long as the examination consists only of items in which no partial scores are awarded, i.e. in which an item can only be scored as having been answered* correctly or incorrectly *by the candidate.

#### 1.2. The elimination procedure and the compensation of disadvantage in flawed items contained in the written component of the state medical examinations

This section presents a short description of the grading method in the written examinations within the German state examination and essentially is a summary and paraphrasing of the explanations given by the IMPP [https://www.impp.de/internet/de/impp-aktuell.html], [1].

On the decision regarding whether an examination has been passed and, if yes, how to grade this examination performance, the Ärztliche Approbationsordnung (German medical licensing regulations ÄAppO, section 14 Written Examination) sets out the following [https://www.gesetze-im-internet.de/_appro_2002/]:

(6) The written examination is deemed to have been passed if the candidate has answered at least 60% of the examination questions posed correctly or if the number of questions answered correctly by the candidate is no more than 22 percent below the average examination performance of the candidates who have participated in the examination for the first time following a minimum study period of two years for the first part the medical examination and five years for the second part of the medical examination.

(7) Performance in the written examination shall be graded as follows:

If the candidate attains the minimum number of correctly answered examination questions required for passing the examination in accordance with paragraph 6, then he or she shall be awarded the grade

– "very good" if he or she answers at least 75%,

– "good" if he or she answers at least 50% but less than 75%, 

– "satisfactory" if he or she answers at least 25% but less than 50%, 

– "pass" if he or she answers none or less than 25% 

of the remaining examination questions correctly.

The grade boundary defined by the 60% criterion shall be known as the "60% threshold". The second criterion ("… 22 percent…") serves to lower the pass threshold in examinations with particularly poor results. For this threshold the term "automatic adjustment clause boundary" (*Gleitklauselgrenze*) is generally used. This is a norm-referenced boundary and depends on the average score achieved in a subgroup of all examination candidates, the "reference group". The reference group is made up of the students who after (precisely) two years or (precisely) five years of study are sitting the examination for the first resp. the second part of the state medical examination for the first time. Consequently the lower of the two thresholds (i. e. the 60% and the automatic adjustment clause threshold) is applied.

Following the examination, all items are once again checked to ensure that they are correct and legitimate on the basis of any appeals/complaints or statistical analyses. During this process it may emerge that an item (i) cannot be correctly answered with the answer options provided, or can be (ii) misinterpreted and/or contain more than one correct answer option. These items are then described as "flawed". The items in group (ii) can thus – whether flawed in content or form – also be answered "correctly".

The flawed questions are "removed from the grading scheme" ("eliminated"); they only count for those candidates who answer the questions justifiably correct (see the judgement of the German Federal Administrative Court of 17.05.1995 [https://www.jurion.de/urteile/bverwg/1995-05-17/bverwg-6-c-8_94/] and the detailed description of elimination and compensation of disadvantage [https://www.impp.de/internet/de/impp-aktuell.html], [1]).

In other words: whilst unanswerable flawed questions are in fact completely excluded from the grading (question group i), flawed items which can be answered correctly (question group ii) are only taken into account for those candidates who have actually answered them correctly.

Example 1: If, for example, of the 320 items in the state examination it is established that in two questions two answers could be considered correct then these two questions are eliminated, i.e. the pass threshold is now 60% of 318 items. This calculation results in a value of 190.8. Thus 191 items ("at least 60%") must be answered correctly in order to pass.

If a candidate answers 190 of the 318 properly formulated items correctly and does not give a correct answer in either of the two eliminated items, then he or she has not passed the examination. 

If the candidate has chosen a correct answer in one of the two eliminated items, then the score from this answer is awarded to him or her. The candidate has then indeed answered 191 items correctly; at the same time, however, the pass threshold increases to 60% of 319 items and thus a value of 191.4, so that 192 correct answers would have been required in order to pass.

Only if both eliminated items are answered correctly would the example candidate have passed: 192 items have been answered correctly, the pass threshold now stands at 192 for 60% of 320 items.

The result of this is that, depending on how the eliminated items have been answered, different items count towards the grades in individual cases and different pass thresholds may apply in individual cases. Thus, where correct answers are given to flawed ("eliminated") items, a "softening" of the minimum standards set out in the licensing regulations (e.g. at least 60% of questions to be answered correctly) is avoided: for example, if at least two possible answers were correct in 10 of the 320 items, and if a candidate also provided a correct answer in all of these items, then the 60% threshold of the 310 properly formulated items is 186. However, since for thies candidate all 320 items are taken into consideration, it is only 186 of 320=58.125%, therefore less than 60%. The pass threshold is therefore defined for thies candidate out of the 320 items: 60% of 320=192. 

#### 1.3. Partial credit scoring

At many medical faculties, however, in examinations items are also used which do not correspond to the classic "one out of five" format ("Type A"), such as for example multiple true false items (frequently also called "Type X" or "Kprim") or "select n answers" from a list of options ("PickN"), for which* partial credit* is awarded for answers which are partially correct.

In this case, adopting the procedure used in the state examinations is not a trivial matter; for example in an item which has been classified as being "flawed in form" but for which a candidate would receive 0.75 points, the question arises as to whether this item should be taken into consideration when calculating the grade or not.

The question is not only significant because multiple-choice questions with partial credit scoring are already being used at a range of faculties, but also because further developments in skills orientated tests of knowledge (written or computer-based examinations) could also require the use of other types of item in which partial knowledge should also be adequately taken into consideration in the grading. Indeed, faculties are explicitly encouraged to develop examinations further in the Masterplan 2020 [2]. On the other hand, the faculties require a certain legal certitude here which is why they have often preferred to proceed in a way which is similar to the state examinations.

In the state examinations themselves, the use of items with partial credit scoring is so far apparently not envisaged; for example, on the one hand it is stated in a comment on a draft of the directive on the new regulations in dentistry education ([3], page 165f) that items of this kind "would permit innovative question and answer formats supported by computer alongside the multiple-choice style question in future", and on the other hand it is "not envisaged […], that a question can have a half-correct answer."

#### 1.4. Objective and Overview

The objective of this paper is to transfer the process of elimination and compensation for disadvantage used in state examinations to examinations which include items with partial credit scoring. 

In doing so we shall consider examinations which consist of items in which only non-negative scores values can be achieved and for which the examination result is composed of the addition of the sum of the scores values achieved in the individual items. There is no requirement that all items must be equally weighted; the maximum achievable score in the various items may thus be different.

In Section 2, the definition of pass and grade boundaries in the state examinations will first of all be described and a rule will be formulated from this which can be transferred to examinations containing items with partial credit scoring. A sub-section will then examine the application of rounding in establishing pass and grade boundaries. 

In Section 3 there will then follow an explanation of which – in the terminology of the IMPP – "eliminated" items shall be taken into account if the procedure proposed here is applied for the individual candidates. The following sections contain a series of "formulae" which are required for a precise presentation. Readers less familiar with mathematical notation should not be put off, we have tried with a series of calculated examples to make the formulae easy to follow.

## 2. Pass and Grade Boundaries

### 2.1. State Examination

In order to describe the regulations of the ÄAppO (in section 14 Written Examination) [https://www.gesetze-im-internet.de/_appro_2002/], the identifiers *B*_S_ for the *mathematical pass threshold* according to the 60% rule and *B*_G_ for the *mathematical pass threshold* according to the automatic adjustment clause (mean of the average examination performances of the reference group – 22%) shall be used. *M* stands for the number of items and *X*_R_ for the mean of the examination performances achieved by the reference group in those items (see table at the end of the article). With these identifiers the following is true (see also the detailed description in [https://www.impp.de/internet/de/impp-aktuell.html], [1])

*B*_S_=0.60 x* M*

*B*_G_=0.78 x* X*_R_

It should be noted here that the mathematical pass thresholds thus defined are not necessarily whole numbers (in the licensing regulations the term "pass threshold" is *not* used). As set out in [https://www.impp.de/internet/de/impp-aktuell.html], the* actual pass threshold* is the smallest whole number which is greater than or equal to the mathematical threshold. If ceil(z) denotes the rounding up function (also known as the "ceiling function"), then the actual pass threshold is ceil(*B*) (for *B*=*B*_S_ or *B*_G_). The mathematical grade boundaries are then

(1) *N*_g_=ceil(*B*)+*g* x (*M*–ceil(*B*))

Whereby the boundary between "fail" and "pass" for *g*=0 (pass threshold), the further boundaries for "satisfactory", "good" and "very good" result in *g*=0.25, 0.50 and 0.75. Note that these boundaries must be *achieved or exceeded* in each case, falling below the boundary even by only a minimal amount results in the poorer grade in each case. The grade boundaries are defined for both *B*_S_ and *B*_G_, the lower of the two thresholds in each case is of significance for the students.

#### 2.2. Pass and Grade Boundaries in Items with Partial Credit Scoring

When partial credit scorig is used, it is not possible to define pass and grade boundaries based solely on the number of "correctly answered examination questions". It would be better to speak of "points" achieved, this formulation permits firstly the use of partial points as well as an unequal weighting of items, amongst other things.

In most degree or examination regulations, absolute and relative pass thresholds ("automatic adjustment clause") are stipulated. If *M* describes the maximum number of points achievable (note that in examinations such as the state examinations, in which one point can be achieved for each item, *M* agrees with the number of items), and *X*_R_ describes the mean score achieved in the examination (if a reference group has been defined, use the mean value in this group), then the absolute pass threshold *B*_S_ and the relative pass threshold *B*_G_ are determined in accordance with the automatic adjustment clause using two constants *c*_S_ or *c*_G_.

*B*_S_=*c*_S_ x* M*

*B*_G_=*c*_G_ x* X*_R_

The value of *c*_S_ is usually 0.6 in medical examinations ("anyone who achieves 60% of the maximum score passes the examination"), *c*_G_ is often 0.78 as in the state examinations ("… anyone achieving not more than 22% … has failed"), for *c*_G_ other values are also used, such as for example at the Medical Faculty of Heidelberg where the value 0.80 is used [4].

The following example shows that the direct application of equation (1) leads to the – in our opinion undesirable – effect that the additional consideration of items with identical properties may first lead to an improvement and subsequently to a lowering of the grade:

Example 2: let us assume that an examination consists of 26 items in which candidates can achieve points in whole numbers from 0 to 4 points in each case. Two items are taken out of the grading scheme.

A candidate achieved 67 points in the 24 correctly set items. The candidate achieved 3 points in each of the two items removed from the grading scheme.

If only the 24 items included in the grading scheme are taken into consideration and the equation (1) used in the state examinations is directly applied, then for the boundary between "pass" and "satisfactory" the result is as follows (a maximum of 4x24=96 points can be achieved):

ceil(*B*)+*g* x (*M*–ceil(*B*))=ceil(0.60 x 96)+0.25 x (96–ceil(0.60 x 96))=ceil(57.60)+0.25 x (96–ceil(57.60))=58+0.25 x (96–58)=67.50

With 67 points the candidate would fall below this boundary, the grade would be "pass".

If we take into account one of the two items removed from the grading scheme, then the result is

ceil(*B*)+*g* x (*M*–ceil(*B*))=ceil(0.60 x 100)+0.25 x (100–ceil(0.60 x 100))=ceil(60.00)+0.25 x (100–ceil(60.00))=60+0.25 x (100–60)=70.00

With 67+3=70 points of a possible 4x25=100 points, the candidate would receive the grade "satisfactory".

If both of the items taken out of the grading scheme, and in which the candidate achieved 3 out of 4 points each, are taken into account, then

ceil(*B*)+*g* x (*M*–ceil(*B*))=ceil(0.60 x 104)+0.25 x (104–ceil(0.60 x 104))=ceil(62.40)+0,25 x (104–ceil(62.40))=63+0.25 x (104–63)=73.25

With 67+3+3=73 points of a possible 4x26=104 the candidate once again only achieves the grade "pass".

Example 2 shows that if 70% of the maximum number of points is achieved, which is normally associated with the boundary between "pass" and "satisfactory", the addition of one item in which 75% of the number of points (3 out of 4 points) was achieved can imply a downgrading. This seems paradoxical to the author. 

##### Formal Definition of the Pass and Grade Boundaries in Items with Partial Credit Scoring

We can achieve a simpler formal definition than equation (1) for the various grade boundaries associated with the pass thresholds *B*_S_ and *B*_G_ with

(2) *N*_g_=*B*+*g* x (*M*–*B*)

which distributes the gap *between mathematical pass threshold and maximum achievable score* into four numerically equal intervals. This is different to equation (1) in that the mathematical pass threshold *B* and not the rounded up actual pass threshold ceil(*B*) is fed into the definition of the grade boundaries.

Using this definition we get around the obvious problem that in an examination with a maximum of 22 achievable points a candidate with 13.5 points would fail, because although the mathematical 60% threshold of 0.6x22=13.2 is exceeded, the candidate would not achieve the rounded up whole number threshold of 14. Further, it is impossible to construct a case similar to example 2 using this definition (the mathematical grade boundaries between "pass" and "satisfactory" calculate out to 67.2, 70.0 and 72.8 using equation (2) for a maximum achievable numbers of points of 96, 100 and 104 points).

The mathematical grade boundaries using equation (2) may be lower than those using equation (1) but never higher. This may cause a discrepancy with the procedure used in the state examination in a particular place. This is conditioned by the formulation "of the remaining examination questions posed" in the ÄAppO which means that in equation (1) ceil(B) must stand instead of B as in equation (2): 

Example 3: Let us assume that an examination consists of 317 items. *The mathematical pass threshold is calculated at 190.2. In the procedure suggested here, the result for the mathematical grade boundary between satisfactory and pass is the value (see equation 2: M=317, B_S_=0.6 x 317=190.2 and with g=0.25) *

190.2+0.25 x (317–190.2)=221.9

If 222 items are answered correctly, the result is “satisfactory”. In the state examination the equivalent grade boundary (equation 1) would be

ceil(190.2)+0.25 x (317–ceil(190.2)=191+0.25 x (317–191)=222.5,

consequently 223 of the items would then have to be answered correctly in order to achieve "satisfactory".

#### 2.3. Rounding

There are, however, justifiable reasons for considering rounding in the definition of pass and grade boundaries:

Example 4: An examination has 17 items in which one whole score can be achieved in each item. According to equation (1) or (2) with the 60% threshold (0.6x17=10.2) at least 11 items must be answered correctly in order to pass. That is 64.7%, making it significantly more than 60%. If only 10 of the items were required in order to pass, this would be less than 60% at 58.8%, but the deviation from 60% would however be significantly lower (1.2% instead of 4.7%).

This also occurs in the state examinations, although with significantly lower percentage variations due to the large number of items. In order to reduce the effect of the "tightening up" of the conditions engendered by the requirement for whole numbers in the examination result achieved (see here also the last paragraph in 1.2), it was for this reason suggested in a draft of the changes to the licensing regulations for dentists [3] in section 34 and section 35 that the mathematical pass threshold be rounded down if the first decimal place is 0 to 4, and rounded up if the first decimal place is 5 to 9. The same applies for the grade boundaries – where the *already rounded pass threshold is* used! These are then described by the equations

(3) B^*^=floor(*B*+½)

*N*_g_=floor(B*+*g* x (*M*–B*)+½)

(B^*^ describes the rounded mathematical pass threshold, floor(z) is the rounding down function, so that the function floor(z+½) rounds down if the first decimal place is between 0 and 4 and up if the first decimal place is between 5 and 9).

The calculation in equation (3) is intended in the draft on the change to the licensing regulations for dentists in practical terms to achieve a lowering of the grade boundaries by half a point in comparison to the current licensing regulations for doctors which uses equation (1). An exception is the boundary point with the precise decimal value of 0.5 which is rounded up.

The effect described in example 2 can also occur with equation (3).

Example 5: The examination consists of 24 items, each with a maximum of 4 points, of which 2 are removed from the grading scheme. The candidate has achieved 3 points in each of these 2 items, in the other 22 items he has achieved 61 points.

Without taking into account the eliminated items, the maximum achievable points for the rounded pass threshold with 4x22=88 are

B^*^=floor(*B*+½)=floor(0.6 x 88+0.5)= floor(52.8+0.5)=53

The boundary between "pass" and "satisfactory" using

floor(B^*^+*g* x (*M*–B^*^)+½)=floor(53+0.25 x (88–53)+0.5)=floor(53+8.75+0.5)=62

Taking into account one of the two eliminated items the result, similarly to the boundary between "pass" and "satisfactory", is 64 points and if both of the items are taken into account then it is 68 points.

As in example 2, the sequential consideration of the two eliminated items with identical properties leads first of all to the candidate failing to achieve the boundary between "pass" and "satisfactory"( 61 points achieved <62), then then achieving it (64 points =pass thresholds 64) and then once again failing to achieve it (67 points <68).

##### Formal Definition of Rounded Pass and Grade Boundaries in Items with Partial Credit Scoring

In items with partial credit scoring, a rounding of the grade boundaries with a similar effect can be achieved by modifying equation (2), thus:

(4)* N*_g_=*B*+*g* x (*M*–*B*)–0.5

An analogue to equation (3) is achieved by further requiring that in order to pass, the pass threshold must not only be achieved but exceeded (the same applies for the grade boundaries). As in example 3, however, this does not result in a complete agreement of (3) and (4).

The change from equation (3) to (4) is justified in the same way as that from equation (1) to (2). The desired reduction by half a point is achieved here by the simple subtraction of the constant 0.5, the requirement that the boundaries are not only achieved but exceeded results from the way in which a case with an exact decimal value of 0.5 is handled (see the last subparagraph). 

## 3. Compensation of Disadvantage in Flawed Items

### 3.1. State Examination

Section 14 of the licensing regulations for doctors [https://www.gesetze-im-internet.de/_appro_2002/] sets out: 

(4) The examination items must […] be checked as to whether they are flawed, measured against the requirements of paragraph 2, sentence 1. If this check shows that individual examination items are flawed then these must not be taken into account when establishing the examination result. The prescribed number of items for the individual examinations […] is reduced accordingly. The evaluation of the written examination […] shall be based on the reduced number of examination items. The reduction in the number of examination items shall not have a negative impact on a candidate.

The last sentence is interpreted in legal terms to mean that flawed items are still to be taken into account in individual cases and will only remain outside of consideration if this is not disadvantageous to the candidate. This means that for each candidate, flawed items are to be considered in such a way that the best possible result for the candidate is achieved, in other words a subset of flawed items is sought which achieves the best possible result for the candidate. This subset is generally not empty.

In the case of the state examinations in which each item can only be marked as correct or incorrect, it can be shown that both for passing and for achieving particular grades, for a particular candidate the best possible result is achieved by the inclusion of all of those eliminated items in which he or she gave a correct answer whilst the other eliminated items are not taken into account.

#### 3.2. Items with Partial Credit Scoring

If the pass and grade boundaries, as defined in sections 2.2 and 2.3 by equation (2) or (4) are applied, it is easy to determine which items should be taken into account.

It must be determined whether an eliminated item in which *m* is the maximum score achievable, *x*_R_ points were achieved at the mean (or the mean of the reference group in the item) and *p* points were achieved by a candidate, should be taken into account for that candidate.

For the grade boundaries derived from B*S*, we must also verify here whether

(5) *g* x* m*+(1–*g*) x* c*_S_ x* m*≤p

and for the grade boundaries of the automatic adjustment clause (with BG as the pass threshold)

(6) *g* x* m*+(1–*g*) x* c*_G_ x* x*_R_≤p

If the inequality condition is met, the item must be taken into account in terms of the respective pass threshold and the grade boundaries derived from it, otherwise not.

Note that the items used to determine the performance of the student thus depend on the grade boundary.

Example 6: In an examination, 102 items are set each offering a maximum of 1 point, one of these items is eliminated due to a defect of form. A student achieves 0.75 points in this item. Depending on whether the student lies at the pass threshold or at the “good”/”very good” boundary, taking the eliminated item into account may be favourable or not for the student.

*If, for example, he achieves 60.50 points and thus just misses the mathematical pass threshold of 60.60, then taking into account the eliminated item has the result that he now has a score of 61.25 meaning that he now lies above the pass threshold of 61.20 points for 102 items (see Table 1* (Tab. 1), *column "Grade boundary fail/pass" and "total score 1"). *

*In the inequality (5) we would need to apply g=0.00, m=1, c*_S_*=0.60 and p=0.75 and*

*g* x* m*+(1–*g*) x* c*_S_ x* m*=0.00x1+(1–0.00) x 0.6 x 1=0.60<0.75=*p*

*If he has achieved 91.00 points in the marked items, thus gaining “very good” (the boundary is 90.90 points at 101 items) then taking into account the flawed item produces a total score of 91.75 which is not sufficient to achieve the boundary of 91.80 points for a “very good” at 102 items (Table 1* (Tab. 1), *column "grade boundary good/ very good" and "total score 2").*

The condition of the inequality (5) is in fact not met here:

*g* x* m*+(1–*g*) x* c*_S_ x* m*=0.75 x 1+(1–0.75) x 0.6 x 1=0.90>0.75=*p*

i.e. the item shall not be included in the grading.

## 4. Discussion

Adopting the approach to elimination and compensation of disadvantage applied in the state examinations in examinations with unequally weighted items and items with partial credit scoring is not difficult to undertake with a similar definition of the pass and grade boundaries. More minor deviations from the definition of the pass and grade boundaries conditioned by the rounding functions in the setting out of the rules of the licensing regulations (see equation 1 vs. 3) are – as shown in example 3 – required to maintain the consistency of the grading scheme.

For determining those eliminated items which must be individually taken into account for a particular candidate in order to achieve or exceed pass or grade boundaries, there are simple conditions which are dependent only on the number of points achieved in the item, the grade boundaries and – if the automatic adjustment clause is applied – the mean number of points achieved in the item. 

The procedure presented is also suitable for application to grading systems other than a division into four grades once the exam has been passed, which is the focus here. If the values for *g* in equations (2) or (4) are broken down finely enough, decimal grades can be awarded, for example.

In general, however, the negative consequences of different pass and grade boundaries in individual cases should be considered. The approach described may well be clear in form, but is not always easy for the students to follow. This is the reason, why, for example, in the grading regulations of the Medical Faculty of Heidelberg [4] it has been set out that points achieved in "flawed" items are awarded to the student as *bonus points*; nevertheless, even if bonus points are awarded, only the pass and grade boundaries derived from the correctly formulated items are applied. The "readjustment" of the pass threshold which takes place in the state examinations is not carried out, the same pass and grade boundaries apply for all candidates.

In consideration of the introduction of innovative examination formats promoted in the Masterplan 2020, it would be welcomed if the rules of any future licensing regulations were formulated from the very beginning in such a way that they were *directly* applicable to examinations containing differently weighted items and/or items for which partial scores are awarded (for example, in the state examination for medicine in Switzerland, half points are awarded in multiple true false items [5]). This also applies in the situation that* for the time* in the written state examinations only items for which no partial scores are awarded are used (see [3]), a generally applicable rule would require no further adjustment if such items were *introduced later*. This would equally secure a unified approach for practical examinations (OSCEs, for example) and a simple application of the grading scheme to examinations in which the pass thresholds are determined using the standard setting procedure and further grade boundaries are mathematically derived from it.

## Notes

Key to symbols used (see Table 2 (Tab. 2))

## Funding

This paper was created as part of the MERLIN II project (01PL17011C) funded by the Federal Ministry for Education and Research.

## Acknowledgements

I would like to express my particular thanks to Dr. H. Shahla of the Institut für medizinische und pharmazeutische Prüfungsfragen for the intensive and constructive discussions.

## Competing interests

The author declares that he has no competing interests.

## Figures and Tables

**Table 1:**
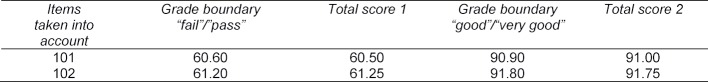
for Example 6: Effect on passing and achieving grade “very good” of taking into account a item with 0.75 points achieved (see text)

**Table 2:**
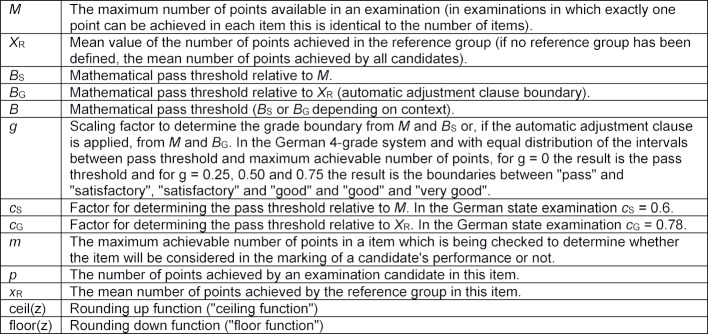
Symbols
